# Increased *Toxoplasma gondii *positivity relative to age in 125 Scottish sheep flocks; evidence of frequent acquired infection

**DOI:** 10.1186/1297-9716-42-121

**Published:** 2011-12-21

**Authors:** Frank Katzer, Franz Brülisauer, Esther Collantes-Fernández, Paul M Bartley, Alison Burrells, George Gunn, Stephen W Maley, Chris Cousens, Elisabeth A Innes

**Affiliations:** 1 Moredun Research Institute, Pentlands Science Park, Bush Loan, Edinburgh, EH26 0PZ, UK; 2 Epidemiology Research Unit, Scottish Agricultural College, King's Buildings, West Mains Road, Edinburgh, EH9 3JG, UK; 3 SAC Consulting, Veterinary Services, King's Buildings, West Mains Road, Edinburgh, EH9 3JG, UK; 4 SALUVET, Animal Health Department, Faculty of Veterinary Sciences, Complutense University of Madrid, Ciudad Universitaria s/n, 28040 Madrid, Spain

## Abstract

*Toxoplasma gondii *seroprevalence was determined in 3333 sheep sera from 125 distinct sheep flocks in Scotland, with the majority of flocks being represented by 27 samples, which were collected between July 2006 and August 2008. The selected farms give a representative sample of 14 400 sheep holdings identified in the Scottish Government census data from 2004. Overall *T. gondii *seroprevalence, at individual sheep level, was determined to be 56.6%; each flock tested, had at least a single positive animal and in four flocks all ewes tested positive. The seroprevalence of sheep increased from 37.7% in one year old stock to 73.8% in ewes that were older than six years, showing that acquired infections during the life of the animals is frequent and that environmental contamination by *T. gondii *oocysts must be significant. The median within-flock seroprevalence varied significantly across Scotland, with the lowest seroprevalence of 42.3% in the South and the highest seroprevalence of 69.2% in the far North of Scotland and the Scottish Islands, while the central part of Scotland had a seroprevalence of 57.7%. This distribution disequilibrium may be due to the spread and survival of oocysts on pasture and lambing areas. A questionnaire accompanying sampling of flocks identified farms that used Toxovax^®^, a commercial vaccine that protects sheep from abortion due to *T. gondii *infection. Only 24.7% of farmers used the vaccine and the vaccine did not significantly affect the within flock seroprevalence for *T. gondii*. The implications for food safety and human infection are discussed.

## Introduction

*Toxoplasma gondii *is the most successful parasitic pathogen world-wide, infecting all warm blooded animals including humans [1]. This protozoan parasite is of great economic importance to the sheep industry. In the UK, *T. gondii *was shown to be the second most commonly diagnosed cause of abortions in sheep (25.4%), after *Chlamydophila abortus *(45.6%), based on data from Veterinary Investigation Diagnosis Analysis (VIDA) for 2009 [2]. The scope for clinical toxoplasmosis is great; in the UK alone there are 14.2 million sheep, of which 6.5 million are breeding ewes [3]. Using average annual incidence rates of clinical toxoplasmosis of between 1% and 2% [4], this would mean that between 65 000 and 130 000 pregnancies are lost annually due to *T. gondii *infection in the UK alone, while in European Union, with a total number of 68.1 million breeding ewes [5] the loss would be between 680 000 and 1 360 000 pregnancies annually.

Most *T. gondii *infections in sheep occur through the ingestion of oocysts, a stage of the parasite, which is very stable and can survive in favourable conditions in the environment for over 12 months, contaminating pasture, feeds and drinking water [6]. Oocysts are produced in the definitive hosts of *T. gondii*, cats, which shed the parasite in their faeces after eating meat, infected with tissue cysts [7]. Intermediate hosts, including sheep and humans, that ingest oocysts are thought to become infected for life, with detectable and persistent IgG antibody levels specific for *T. gondii *and this has aided the assessment of infection levels in many host species [8].

Abortions in sheep due to *T. gondii *are associated with a primary infection during the first or second trimester of gestation, while primary infection in the later stages of pregnancy leads to the birth of congenitally infected lambs, which are relatively rare [9,10]. Infected animals develop an effective immune response, which will protect against disease in subsequent pregnancies [11]. These studies and others showed that vaccination may be a feasible option to control disease and lead to the development of a live vaccine preparation. Toxovax^® ^was licensed in 1988 in New Zealand and was launched in 1992 in UK and Ireland [12]. Toxovax^® ^consists of a live attenuated *T. gondii *strain (S48) that will not cause a persistent infection [13,14]. The efficacy and safety of this vaccine were tested by Intervet B.V. in collaboration with the Moredun Research Institute in Scotland, where it was shown that a single vaccine dose will induce an effective immune response that will persist for at least 18 months without further challenge [15].

Human congenital infections with *T. gondii *are very similar to those seen in pregnant sheep but there are no licensed vaccines for humans that protect against toxoplasmosis. Another difference is the main transmission route by which humans may become infected [16]. A multicentre epidemiologic study among pregnant women in Europe identified ingestion of raw or undercooked meat/meat-products, containing tissue cysts, as the major source of *T. gondii *infection, while infection through oocysts, from environmental contamination, plays only a minor part [17].

In 2007, 1851 cases of *T. gondii *infection in humans were clinically diagnosed and reported by 18 European Union member states, 149 of which originated from the United Kingdom [18], giving an incidence rate of 0.83 reported clinical cases per 100 000 people within Europe. Although this incidence seems low, this does not express the severity of the potential consequence of infection, which could be abortion or the birth of congenitally infected babies, which may suffer from learning disabilities, requiring assistance throughout life; these congenitally infected children are likely to develop ocular toxoplasmosis later in life. Therefore the resulting disease burden, due to *T. gondii *infection, was rated higher than that of any other food borne pathogens in Europe, by the National Institute of Public Health and the Environment (RIVM) of The Netherlands [19,20]. In another recent report by the RIVM, based on calculations of the long-term socio-economic impact of emerging zoonotic pathogens relevant for The Netherlands, *T. gondii *was ranked as the second most important pathogen due to its high disease burden [21].

The European food safety authority recommended in 2007, that there is a need to improve data collection on surveillance and monitoring of *T. gondii *in animals and food products for human consumption, in order to better evaluate the disease risk of toxoplasmosis in member states [22]. There is no current data on the prevalence of *T. gondii *in food animals in the UK and very little is known about environmental contamination levels of *T. gondii *oocysts. Existing prevalence figures are only regional and predate the licensing of Toxovax^® ^in the UK. In this study we set out to ascertain how frequent *T. gondii *infections are within a representative section of Scottish sheep flocks, when sheep become infected and how widely Toxovax^® ^is used.

## Materials and methods

### Study design and serum collection

The serum samples for the *T. gondii *prevalence study stem from a national study, aimed to assess the prevalence of different endemic sheep diseases in Scotland, conducted between July 2006 and August 2008 [23]. The sample frame comprising 825 sheep holdings was generated on the basis of Scottish Government census data from June 2004 and was stratified by three separate regions (South (i.e. south of Glasgow and Edinburgh), Central (i.e. Aberdeenshire, Perthshire and Argyll) and North (i.e. Highlands and Islands) consisting of 40, 35 and 50 farms, respectively) to take account of the distribution of sheep of different Scottish regions. Flocks with at least 50 breeding ewes were eligible to take part in the study; on farms that had multiple flocks, information was gathered only for one flock. Study farms were initially contacted by mail and then by telephone to identify farmers willing to participate in the study. On 125 farms information was gathered on general farm characteristics, management practices and animal health during a face-to-face interview; the standardised questionnaire had been designed with the help of sheep specialists and was pre-tested on 5 sheep owners. At the end of the interview farmers were given a printout of the questionnaire for a crosscheck to ensure optimal data quality. A postal follow-up questionnaire including specific questions on the use of Toxovax^®^, a commercially available vaccine against toxoplasmosis, was also conducted. Age and breed were recorded for typically 27 sheep per flock from which serum samples were taken. The samples were sent to the laboratory by next day delivery at ambient temperature and serum was separated from clotted blood and stored at -20 C until tested.

### Serology

An ELISA was used to detect IgG antibodies to *T. gondii *in sheep sera as described by Buxton and colleagues [24]. This ELISA used a water-soluble antigen fraction of *T. gondii *tachyzoites of the S48 strain [25]. Data was expressed as percentage positivity in comparison to a standard, high titre, positive control serum obtained from an experimentally infected animal. Optical density (OD) readings, resulting in a percentage positivity under 25% were considered to be negative; percentage positivity values of 25% and under 30% were classified as borderline and percentage positivity values of 30% and above were interpreted as positive [24]. Sera were tested in duplicate and results that did not match or borderline results were repeated.

### Statistical analysis

Data were described using graphs and boxplots produced in MS Office Excel (version 2007) and R (version 2.12.0). Univariate tests of equality were performed at animal level (age) and flock level (geographic region, management practices). Individual animals were treated as independent. Differences between strata of varied management practices and geographic regions were assessed by Wilcoxon-Mann-Whitney test; confidence bands were calculated using bootstrapping methods [26]. Linear regression models were used to assess the association of age with serostatus of individual animals as well as geographic latitude with within-flock seroprevalence. Variables identified at the univariate stage of analysis were included in multivariate regression models and non-significant variables were removed in a stepwise fashion. For variables with multiple categories, all categories were maintained in model if one category proved significant. The final model selection was based on the Akaike information criterion (AIC), as well as the plausibility to affect seroprevalence of explanatory variables.

## Results

### *T. gondii *seroprevalence

**Figure 1:**
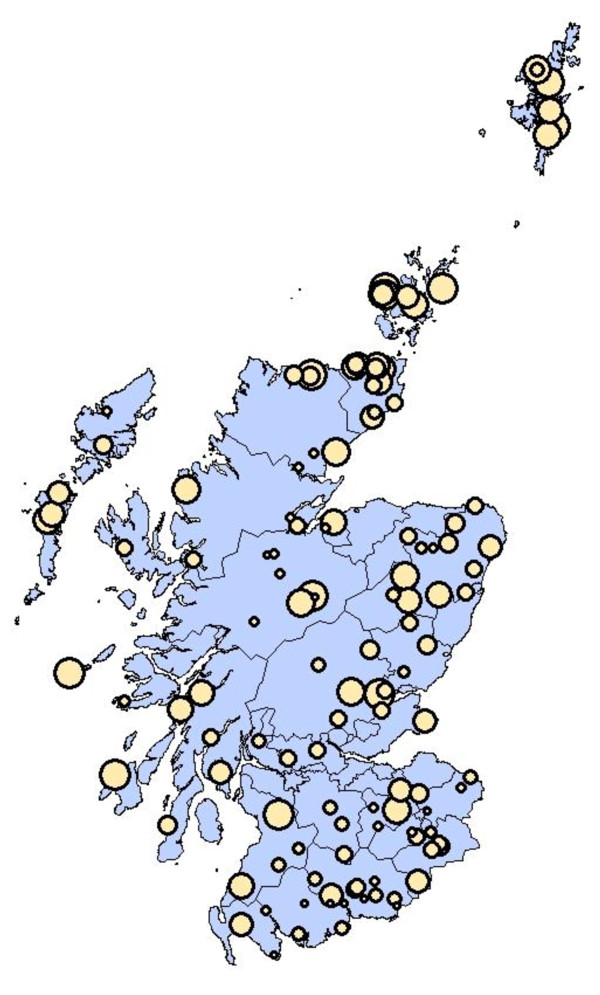
**Geographic distribution of 125 study farms across Scotland and their *T. gondii *seroprevalence**. The circles map the location of the study farms, according to their post code. The size of a circle is directly proportional to *T. gondii *seroprevalence within the corresponding sample of the sheep flock. All farms had at least a single seropositive animal and in four farms all out of a sample of 27 animals tested positive for *T. gondii *antibodies.

A total of 3333 sheep serum samples were collected from 125 farms (between 25 and 31 samples per farm, with the majority of farms (*n *= 109) being represented by 27 sera), spread across Scotland to give a representative serum panel. A total of 1712 serum samples tested positive for *T. gondii *antibodies, while 310 samples gave a borderline result and 1311 sera were negative. At animal level, this resulted in an overall seroprevalence of 51.4%, counting the borderline results as negative. Due to the difficulty of interpreting the borderline case results it was decided to exclude them from subsequent statistical analyses, resulting in a *T. gondii *prevalence of 56.6%. All study farms had *T. gondii *seropositive sheep, varying from single animals (3.7%; one out of 27 animals) on four farms to farms (*n *= 4) where all sheep sampled were positive (100%). Figure 1 presents the geographical distribution of the study farms across Scotland and their *T. gondii *seroprevalence.

### Effect of vaccination on *T. gondii *seroprevalence

The follow-up questionnaire data were analysed in order to evaluate if the use of Toxovax^®^, a commercially available vaccine against toxoplasmosis, had a significant impact on the seroprevalence shown above. Questionnaires were returned by 93 of the 125 farmers, with an equal response rate across the different regions. The vaccine was used by 22 of the 93 farmers that responded (24.7%). In most cases the farmers used the vaccine on a yearly basis (*n *= 16) to vaccinate their replacement stock, while the remaining farmers used the vaccine less frequently. The vaccine uptake was about equal across the country, when divided into three regions: South 8/32 (25.0%), Central 6/26 (23.1%) and North/Islands 8/35 (22.9%). The median within flock seroprevalence for *T. gondii *on farms without vaccination was 55.5% (95% CI: 50.0, 65.2%) and on farms with vaccination 59.2% (95% CI: 53.8, 82.7%). Statistical comparison revealed no significant difference of median within-flock prevalence between flocks with and without vaccination (*p*-value = 0.29). Consequently the full data set (*n *= 125) was used for further analysis.

### Age effect on *T. gondii *seroprevalence

**Figure 2:**
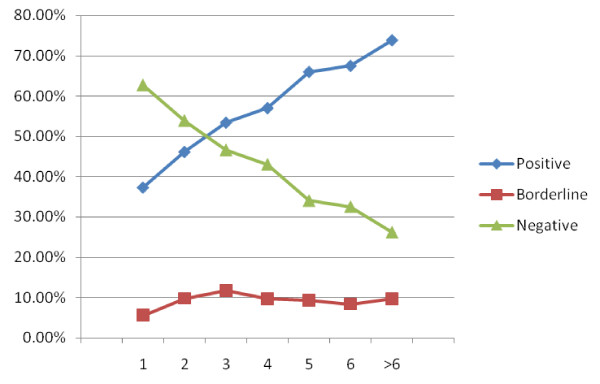
***T. gondii *seroprevalence in sheep according to age of animal**. The percentage of sheep being *T. gondii *seropositive increased from 37.3%, in animals that are one year old, to 73.8% in animals that are more than 6 years old, while the number of borderline samples stayed fairly unchanged for the different age groups. Borderline cases were excluded from the calculation of the age-related *T. gondii *seroprevalence.

The age of 3171 sheep was recorded. This allowed the association between seroprevalence and age to be assessed to determine if an increased cumulative chance of environmental exposure to *T. gondii *will be reflected in increased seroprevalence in older animals. Seropositivity for *T. gondii *increased with age from 37.7%, for sheep that were about 1 year old, to 73.8%, for sheep that were older than 6 years. The increasing *T. gondii *seroprevalence with age is illustrated in Figure 2, while the actual figures and percentages are shown in Table 1. The regression coefficient for age predicting OD reading was estimated to be 3.1 (standard error: 0.38, *p*-value: < 0.001). The frequency of borderline cases (*n *= 309) per age group ranged from 5.5% to 11.7% of sheep per age group but no age dependency was observed (Figure 2).

**Table 1:** Seropositivity for *Toxoplasma gondii *in sheep according to animal age.

Age of Sheep	Numbers of Animals			Percentage of Animals		
	
	Positive	Negative	Borderline	Positive*	Negative*	Borderline
1 year	76	128	12	37.3%	62.7%	5.6%
2 years	247	289	58	46.1%	53.9%	9.8%
3 years	351	307	87	53.3%	46.7%	11.7%
4 years	340	257	64	57.0%	43.0%	9.7%
5 years	292	151	45	65.9%	34.1%	9.2%
6 years	209	101	28	67.4%	32.6%	8.3%
> 6 years	104	37	15	73.8%	26.2%	9.6%

Total	1619	1270	309	56.0%	44.0%	9.7%

* Borderline results were excluded for calculation of seroprevalence in age cohorts

### Regional variation in *T. gondii *prevalence

**Figure 3:**
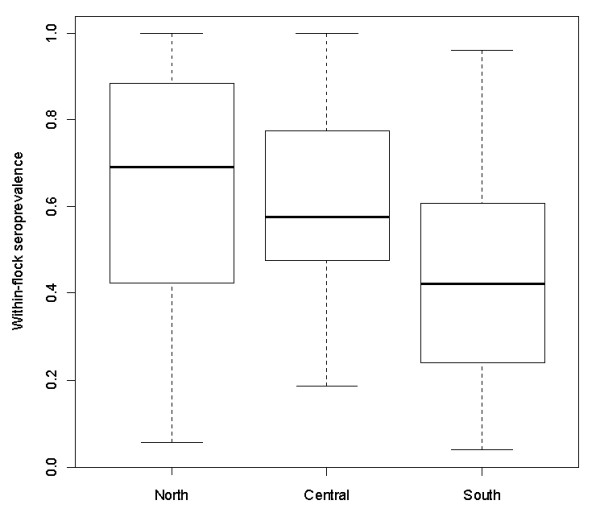
**Within-flock *T. gondii *seroprevalence in study farms stratified by geography**. Southern (i.e. south of Glasgow and Edinburgh), central (i.e. Aberdeenshire, Perthshire and Argyll) and northern (i.e. Highlands and Islands) regions of Scotland. The boxplots illustrate minimum and maximum, first and third quartiles as well as median.

*Toxoplasma gondii *seroprevalence was calculated for three separate regions of Scotland. The lowest *T. gondii *seroprevalence was seen in the south of Scotland, where the median within-flock prevalence was 42.3%, which increased to 57.7% in central Scotland and was at its highest with 69.2% in North/Islands as shown in Figure 3, whereby the difference between south and north was statistically significant (*p*-value: < 0.001).

### Risk factors

**Figure 4:**
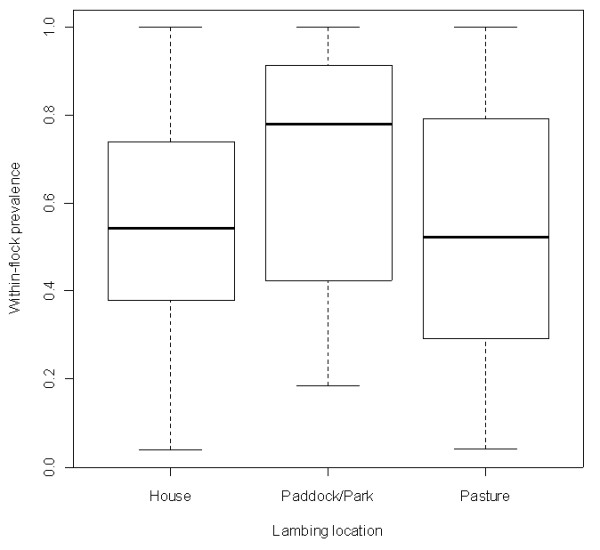
**Within-flock *T. gondii *seroprevalence on 125 study farms stratified by lambing location**. Ewes housed for lambing (House), lambing on restricted area outside (Paddock/Park) and lambing on pasture (Pasture). The boxplots illustrate minimum and maximum, first and third quartiles as well as median.

Univariate analysis identified several risk factors associated with lambing and biosecurity as being associated with higher and lower *T. gondii *seroprevalence values. Farms where lambing took place on pasture (*n *= 51) had a median within-flock seroprevalence of *T. gondii *of 52.2%; farms where animals were housed during lambing (*n *= 56) had a within-flock prevalence of 54.4% but farms where sheep were kept in paddocks or "parks" during lambing (*n *= 18) had a significantly higher median within-flock prevalence of 78.3%. (*p*-value = 0.050). These results are illustrated in Figure 4. Grazing of sheep together with sheep from other farms on common pasture was also identified as a potential risk factor (*p*-value = 0.019). The mean within-flock prevalence for farms, where sheep had no contact with sheep from other farms, (*n *= 105) was 52.2%, while farms, where sheep shared pasture with sheep from other farms, (*n *= 20) had a median within-flock prevalence of 72.6%. Type of fencing (i.e. double versus single fence) of the pasture did not have any significant effect on median within-flock *T. gondii *seroprevalence but farms that were contained by a single boundary (*n *= 70) had a lower median within-flock seroprevalence (51.0%) than farms with multiple boundaries (*n *= 55; 62.5%, *p*-value = 0.027).

Multivariate analysis with region, common pasture, mean sheep age, farm boundary and lambing location as explanatory variables identified mean sheep age and boundary consistently as significant risk factors for within-flock *T. gondii *seroprevalence. The best fitting model included region as an explanatory variable; however, deemed a proxy for geographic differences in flock management, further models were run without region. Common pasture consequently was identified as significant factor, whilst lambing location made no significant additional contribution to explain the variability observed in seroprevalence. Sheep breed was not found to be a significant factor for *T. gondii *seroprevalence.

## Discussion

The results from this Scotland wide study showed an overall seroprevalence for *T. gondii *in sheep of 56.6%. Previous seroprevalence studies in Scotland, conducted 20 years ago, only examined animals from specific regions where the prevalence of *T. gondii *in sheep from locations around Glasgow was found to be 26.2% [27].

Results from our study showed an increase in *T. gondii *seroprevalence with age confirming that a major source of infection for sheep is likely to be through the consumption of sporulated oocysts from the environment. This trend has also been reported by other authors where seroprevalence to *T. gondii *in market weight lambs was found to be only about half that of adult ewes [12], reflecting an increase in seropositivity with animal age. Further studies looking at development of specific antibodies in sheep, as an indicator of exposure to *T. gondii*, have shown that there is an increase in seroprevalence associated with age indicating that there is extensive environmental contamination with *T. gondii *oocysts and that most infections in sheep occur following birth [28-30]. These studies taken together with the extensive study reported in this paper do not support the hypothesis, based on a more limited data set, that vertical transmission of *T. gondii *is significant in sheep flocks [31]. This direct correlation of increasing age and sero-prevalence has also been described in humans [32-34].

During *T. gondii *infection animals mount a humoral immune response which can be detected by measuring immunoglobulin G (IgG) antibody levels. Following an initial infection, antibody levels are expected to stay elevated for the lifetime of the animal, because the immune response of infected animals does not completely clear the parasite and animals stay persistently infected. However, recent longitudinal serology based studies have shown that *T. gondii *IgG antibody levels may drop in some infected animals leading to false negative results [35,36]. One of these studies followed 524 sheep for one year and *T. gondii *antibody levels were measured at 4 different time points. Around 10% of the animals had negative test results following a previously positive result which raises issues about the sensitivity of some of the serological assays used to measure *T. gondii *antibodies and also the persistence of the elevated antibody titres following infection.

This study revealed a significant positive correlation of increasing *T. gondii *prevalence and age of animals tested, reflecting an accumulation of *T. gondii *infection throughout life. This study confirms that sheep in Scotland are frequently exposed to *T. gondii *oocysts and that horizontal transmission is a significant route of infection. This observation provides further support to previous papers stating that horizontal rather than vertical transmission is the main cause of ovine abortion [6,9,10,12,37]. Smaller increases in *T. gondii *prevalence, observed between the older age groups are likely to represent a decreasing number of naïve animals, in these groups, that are still able to become infected. This may yet be further confounded by some persistently infected animals, without recent *T. gondii *challenge becoming antibody negative as seen in longitudinal seroprevalence studies [35,36]. This effect will result in a reduction of detection of infected animals, which will particularly affect the older age groups. A reason, why the higher rate of *T. gondii *seroconversion is seen in the younger age groups, maybe due to the use of Toxovax^®^. Analysis of the questionnaire data revealed that most farmers tend to vaccinate replacement ewes, i.e. young animals. The vaccine consists of a live attenuated strain that does not cause persistent infection [11]. It induces humoral and cell mediated immune responses that will protect sheep very effectively for more than 18 month against *T. gondii *induced abortions without the need for booster vaccination or field challenge [11]. However, detectable antibody levels may not persist for more than six month without subsequent challenge [38]. Therefore, an increased rate of seroconversion is likely to be seen in the younger age groups. However, this effect is not long lived, without field challenge, and did not result in a significant difference in mean within flock difference for vaccinated and non-vaccinated sheep due to mixed ages of animals tested on each farm.

The results of this study show that there is a gradient of *T. gondii *seroprevalence across Scotland, where the South has a significantly lower mean within-flock seroprevalence (36.8%) than the North/Islands (60.5%), with the Central part of Scotland having an intermediate seroprevalence (54.8%). This result is independent of sheep breed, which was not significantly associated with *T. gondii *infection. The observed infection gradient is different to trends seen in other European studies, in France [39] and Finland [40], where studies have shown higher seroprevalence figures for the south of the countries and lower figures for the north. The French study observed an increasing prevalence gradient from north-western to southern France in sheep and explained this due to more favourable conditions to oocyst survival [39]. The Finnish study reported a geographical gradient in *T. gondii *prevalence for sheep and moose, where the seroprevalence in both host species was lower in the North and higher in the South-Western part of Finland. The authors suggested that this observation may reflect areas of habitation and therefore cat density because *T. gondii *oocysts are very unlikely to survive the severe Finnish winters. It is unlikely that the figures obtained for Scotland reflect cat density or areas of habitation alone but are also likely to involve minimum temperatures during winter, which for the UK does not only depend on a North-South gradient but may depend on altitude and closeness to the coast and other large bodies of water, which do not freeze during winter and may aid oocyst survival during winter. In the absence of severe winters in the UK, Ireland and New Zealand, it has been speculated that oocyst may survive in the environment for up to 18 month due to the mild and humid climates in these countries [40]. Climate change predictions of increasing temperatures, drier summers, and wetter winters have lead to the speculation that sporulated oocyst survival is likely to increase, leading to an increase in *T. gondii *prevalence in intermediate and final hosts [41]. The latter study focused on the effect climate change on *T. gondii *prevalence in Europe and predicted an increase in human toxoplasmosis cases that will be higher in the South of England with only modest increases in the North of England and Scotland. However, *T. gondii *prevalence figures in this survey would indicate that sheep in the North of Scotland and the Scottish Islands are either more susceptible hosts for *T. gondii*, which seems unlikely, since no breed effect was identified. An alternative reason is that there is more environmental contamination by viable oocysts, which may reflect more favourable conditions for oocysts survival or alternatively higher cat densities.

The analysis of the questionnaire has shown that the practice of lambing in paddocks is a potential risk factor leading to increased seropositivity in sheep flocks, which could be explained by the proximity of these paddocks to farm buildings and access by cats, which in turn may lead to elevated levels of oocysts present in the paddocks as a source of infection for the sheep flock. A previous study identified grazing by lambs in close proximity to farms as potential risk factor for *T. gondii *infection and the authors recommended that lambs should not be grazed near farms to lower exposure to oocysts shed by cats [42]. Paddocks, in this study, are likely to represent a similar risk factor. Variation in lambing practices and cat densities may explain differences in *T. gondii *flock prevalence figures for farms are geographically very close.

The questionnaire results also highlighted that there is a relatively low (24.7%) uptake of the Toxovax^® ^vaccine, which protects ewes very effectively against *T. gondii *associated abortion. All farms had sheep that were seropositive indicating that all flocks had exposure to *T. gondii *and the potential of experiencing abortions induced by this parasite. Analysis of the questionnaire data did not allow identification of reasons for the low vaccine uptake by farmers but it could be speculated that only farmers that had previous abortion problems that were caused by *T*. *gondii *used the vaccine. The low uptake of this vaccine may also explain why *T. gondii *is still the second most frequently detected abortifacient in ovine abortions, according to current VIDA reports [2].

The high *T. gondii *seroprevalence in Scottish sheep flocks indicate that a significant number of persistently infected animals will enter the food chain. A recent survey identified that eating undercooked meat is not commonly seen as a risk factor for *T. gondii *infection by people, only 30% of people asked, were aware of this risk, while the majority recognised cats as risk [43]. In Europe it has been well documented that a major source of *T. gondii *infection for humans is ingestion of meat or meat products containing *T. gondii *tissue cysts that have not been inactivated, either by cooking or freezing [16,17,22]. Vaccines that would have great potential beneficial to both animal and public health would be those that reduce or stop shedding of oocysts by cats [44,45] or reduce *T. gondii *cyst formation in food animal species [11,46,47], particular targets would be sheep and pigs [12,43,46-48], while cattle seem to pose a lower risk for transmission of *T. gondii *via infected meat [49].

The prevalence figures described in this study demonstrate that *T. gondii *is widely distributed across sheep flocks in Scotland, and that the within-flock prevalence is not the same across Scotland but that it is higher in the North and the Islands and significantly lower in Southern Scotland. An increase in seroprevalence was associated with increasing animal age, indicating that acquired infection in life due to oocyst consumption is a major route of transmission. The seroprevalence data also indicates that contamination of the environment with oocysts is high in Scotland and that sheep meat if undercooked may pose a significant risk of *T. gondii *transmission to people.

## Competing interests

The authors declare that they have no competing interests.

## Authors' contributions

FK and EAI conceived the study and participated in its design and coordination; CC and GG were responsible for the selection of farms, and obtaining serum samples. ECF, PB, AB and SWM performed the serological (ELISA) analysis. Statistical analysis, Questionnaire design and analysis was carried out by FB. FK, EAI and FB drafted the final version of the manuscript; with inputs form all other authors. All authors read and approved the final manuscript.
